# Ion‐Pair‐Tuned Ionogels for Broad‐Range Linear Pressure Sensing

**DOI:** 10.1002/advs.202524195

**Published:** 2026-03-12

**Authors:** Hyeonseo Joo, Tianhao Yu, Yumin Dai, Seokkyoon Hong, Axel González Cornejo, Tristan Michael Long, Sang Mok Park, Pete S. Kollbaum, Edgar Bolívar‐Nieto, Young L. Kim, Dong Rip Kim, Chi Hwan Lee

**Affiliations:** ^1^ Weldon School of Biomedical Engineering Purdue University West Lafayette Indiana USA; ^2^ School of Mechanical Engineering Hanyang University Seoul Republic of Korea; ^3^ School of Mechanical Engineering Purdue University West Lafayette Indiana USA; ^4^ School of Materials Engineering Purdue University West Lafayette Indiana USA; ^5^ Department of Aerospace and Mechanical Engineering University of Notre Dame Notre Dame Indiana USA; ^6^ School of Optometry Indiana University Bloomington Indiana USA; ^7^ Elmore Family School of Electrical and Computer Engineering Purdue University West Lafayette Indiana USA; ^8^ Birck Naotechnology Center Purdue University West Lafayette Indiana USA; ^9^ Center for Implantable Devices Purdue University West Lafayette Indiana USA

**Keywords:** capacitive pressure sensors, healthcare monitoring, ionogels, ionic gels, intraocular pressure sensors, prosthetics, soft bioelectronics

## Abstract

Ionogels combine the mechanical softness of polymers with the ionic conductivity and nonvolatility of ionic liquids, offering a versatile platform for wearable electronics and sensing applications. In particular, their high deformability and ionic responsiveness make them attractive dielectric materials for capacitive pressure sensors. However, conventional ionogels often exhibit dielectric saturation and nonlinear responses at elevated pressures, limiting their usable operating range. Here, we report ion‐pair‐tuned ionogels that balance ionic mobility and polarizability to mitigate dielectric saturation and broaden the linear sensing range up to the megapascal level while maintaining high sensitivity. To validate broad‐range functionality—from subtle physiological pressures to large mechanical loads—we integrate the ionogels into intraocular pressure sensors and prosthetic interface monitors, representing low‐ and high‐pressure regimes. These demonstrations establish ion‐pair tuning as an effective strategy for achieving broad linear sensing performance in wearable pressure sensors.

## Graphical abstract

Ionogels engineered through ion‐pair tuning extend the linear sensing range of capacitive pressure sensors. By balancing ionic mobility and polarizability within the polymer matrix, this approach mitigates dielectric saturation and maintains a stable pressure response across a broad operating range. Their practical utility is further demonstrated through integration into intraocular and prosthetic pressure‐sensing platforms.

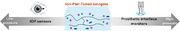

## 1 Introduction

Continuous and accurate pressure monitoring is essential for wearable biomedical sensors [1, 2, 3, 4, 5, 6] that must operate across both low‐pressure conditions—such as intraocular pressure (IOP) assessment [7, 8, 9] (≈1–5 kPa)—and high‐pressure environments, including prosthetic interface management [10, 11, 12] (up to ≈350 kPa). These diverse applications require devices that are soft, conformable, and mechanically durable under repeated deformation while maintaining stable and reliable electrical responses over extended use [13, 14, 15, 16, 17, 18, 19, 20]. Among various sensing approaches, capacitive pressure sensors have emerged as a leading candidate because they offer low power consumption, simple device architectures, and compatibility with flexible dielectric materials [21, 22, 23, 24]. In practice, however, wide‐range pressure monitoring often depends on separate sensors or dielectric formulations optimized for either low‐ or high‐pressure regimes, complicating calibration and device integration [25, 26]. A single dielectric material capable of delivering reliable and linear responses across the full pressure spectrum would greatly simplify system design and enable continuous, uninterrupted sensing. Yet existing materials frequently become constrained at elevated pressures, where the pressure–capacitance response transitions into nonlinear or saturating behavior [27, 28], reflecting a limited ability of the dielectric to sustain further polarization under increasing compression, thereby limiting accurate and calibration‐free operation across broad pressure ranges. Overcoming dielectric saturation while preserving linearity therefore remains a central challenge for advancing capacitive pressure sensors.

A wide range of dielectric materials has been developed to improve the sensitivity and linear range of capacitive pressure sensors, yet each class presents intrinsic trade‐offs among flexibility, permittivity, and stability [23, 29]. Inorganic oxides offer high dielectric constants but lack mechanical compliance, making them unsuitable for soft wearable platforms [30]. Elastomeric polymers such as polydimethylsiloxane (PDMS) [31, 32], thermoplastic polyurethane (TPU) [33, 34, 35, 36], and the commercial VHB acrylic elastomer (3M) [37] provide flexibility and resilience but possess intrinsically low permittivity, limiting sensitivity. Strategies such as incorporating high‐permittivity fillers [38, 39] or introducing microstructured geometries [40, 41] can improve responsiveness but often lead to nonlinear deformation and increased fabrication complexity. Hydrogels, composed of water‐based ionic networks, exhibit exceptional softness and ionic conductivity but suffer from evaporation and degradation over time, resulting in poor environmental durability [42, 43, 44]. These limitations motivate the pursuit of ionogels as next‐generation dielectric materials that combine the mechanical softness of polymers with the ionic conductivity and nonvolatility of ionic liquids (ILs).

Ionogels, consisting of ILs immobilized within polymer networks, enable the formation of electric double layers (EDLs) at electrode interfaces, facilitating rapid polarization and stable capacitive responses under deformation [45, 46, 47]. These features make ionogels well‐suited for sensing applications that require both mechanical compliance and reliable dielectric performance [48, 49, 50, 51]. Nonetheless, many ionogels tend to exhibit nonlinear and saturating behavior at high pressures, likely arising from unbalanced ion transport and localized charge accumulation [52]. These effects limit consistent and accurate pressure sensing across broad pressure ranges, especially when transitioning between physiological and mechanical regimes.

Here, we report ion‐pair‐tuned ionogels composed of 1‐ethyl‐3‐methylimidazolium dicyanamide ([EMIM][DCA]) and 1‐ethyl‐3‐methylimidazolium bis(trifluoromethylsulfonyl)imide ([EMIM][TFSI]), introduced as a materials‐level dielectric design strategy to regulate ionic polarization behavior. Ion‐pair tuning reduces charge accumulation and delays dielectric saturation, enabling an extended linear pressure‐sensing range without structural or geometric modification. We demonstrate its versatility through two representative wearable sensing platforms: IOP monitoring, which involves low physiological pressures (≈1–5 kPa), and prosthetic adaptor interface sensing, which typically operates under high mechanical pressures (up to ≈350 kPa). These demonstrations confirm the broad‐range linear sensing capability of ion‐pair‐tuned ionogels, highlighting their utility as reliable dielectric materials for wearable pressure sensors.

## 2 Results and Discussion

### 2.1 Material Design and Ionic Composition Strategy

**FIGURE 1:**
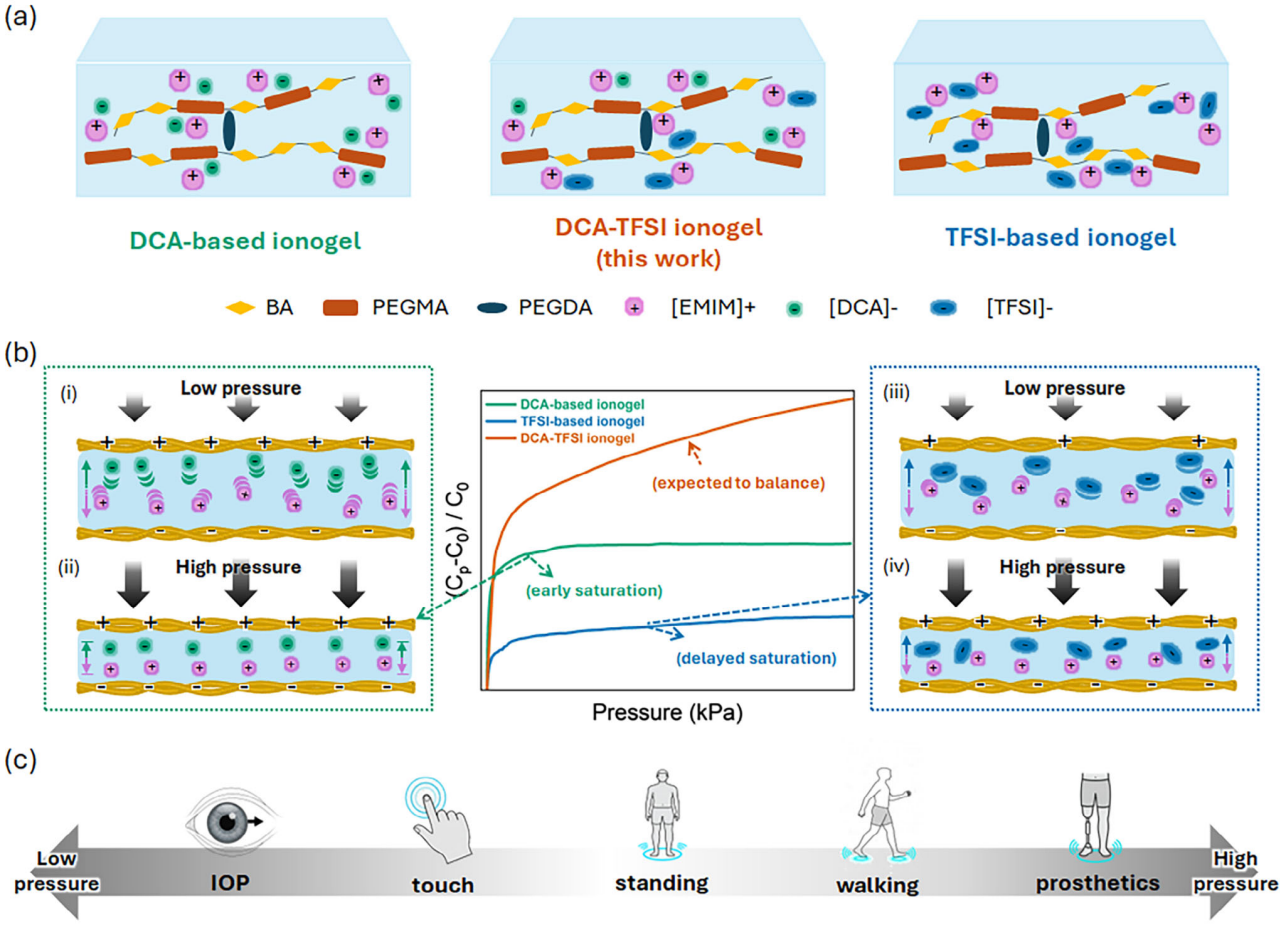
(a) Schematic illustration of DCA‐based, TFSI‐based, and DCA‐TFSI ionogels with different anionic species. (b) Pressure‐dependent dielectric behavior showing early saturation in DCA‐based, delayed saturation in TFSI‐based, and balanced response in DCA‐TFSI ionogel. (c) Representative pressure regimes for healthcare monitoring ranging from low‐pressure IOP sensing to high‐pressure prosthetic interface applications, illustrating wide‐range pressure evaluation using a single material platform.

To achieve reliable and linear capacitive sensing across a wide pressure range, we design an ionogel‐based dielectric that preserves both stability and sensitivity without relying on geometric microstructuring or repeated calibration. We select ionogels as the foundational matrix because they intrinsically combine ionic conductivity, mechanical softness, and environmental robustness (Table S1). As illustrated in Figure 1a, 1‐ethyl‐3‐methylimidazolium dicyanamide ([EMIM][DCA]) contains small, highly mobile anions that facilitate rapid charge redistribution, whereas 1‐ethyl‐3‐methylimidazolium bis(trifluoromethylsulfonyl)imide ([EMIM][TFSI]) contains bulky, strongly polarizable anions that stabilize the electrostatic environment under compression. We hypothesize that incorporating these two ionic species within a single polymer network can redistribute polarization contributions across different pressure regimes, thereby improving dielectric linearity across a broad pressure spectrum. This design is motivated by the distinct physicochemical characteristics of DCA^−^ and TFSI^−^ anions, including differences in ion size, charge delocalization, and mobility, which are known to influence ion transport and polarization behavior in ionic systems [53, 54]. These physicochemical contrasts guided the deliberate selection of the DCA^−^/TFSI^−^ pair, enabling distinct polarization dynamics while maintaining a common EMIM cation framework to isolate anion‐specific effects. The detailed chemical composition and ultraviolet (UV) curing process are illustrated in Figure S1, which depicts the monomers—benzyl acrylate (BA), poly(ethylene glycol) methyl ether methacrylate (PEGMA), and poly(ethylene glycol) diacrylate (PEGDA)—together with the two ILs, [EMIM][DCA] and [EMIM][TFSI], incorporated into the polymer matrix. In this study, ionogels incorporating a single IL are referred to as the DCA‐based and TFSI‐based ionogels, whereas the ion‐pair‐tuned material is referred to as the DCA–TFSI ionogel.

The resulting ionogels exhibit distinct dielectric behaviors depending on their ionic‐liquid composition (Figure 1b). The DCA‐based ionogel shows a steep initial capacitance increase followed by early saturation, consistent with rapid polarization of the small DCA^−^ anions under compression. In contrast, the TFSI‐based ionogel displays a slower, more gradual capacitance increase because the bulkier TFSI^−^ anions stabilize the electrostatic environment and delay polarization. The ion‐pair‐tuned DCA–TFSI ionogel exhibits an intermediate and more balanced capacitive response, reflecting the complementary contributions of the two anions. While demonstrated using the DCA–TFSI system as a model pair, the proposed ion‐pair tuning concept is guided by physicochemical contrast in ion transport and association. Ion pairs exhibiting similar asymmetry may display comparable pressure‐dependent polarization redistribution, although the quantitative dielectric response will remain system‐dependent. To assess performance under realistic loading conditions, we selected two representative pressure regimes (Figure 1c). The intraocular pressure (IOP) model captures small physiological pressure fluctuations and enables evaluation in the low‐pressure range, while the prosthetic adaptor interface model involves substantial mechanical loading and repeated high‐pressure cycling, providing a platform to examine stability and durability under more demanding conditions. Together, these two regimes enable a comprehensive evaluation of pressure‐dependent linearity and operational robustness across a broad pressure spectrum, demonstrating the capability of a single material platform to operate reliably under widely different pressure conditions.

### 2.2 Ion‐Pair‐Tuned Composition and Device‐Level Parameters

**FIGURE 2:**
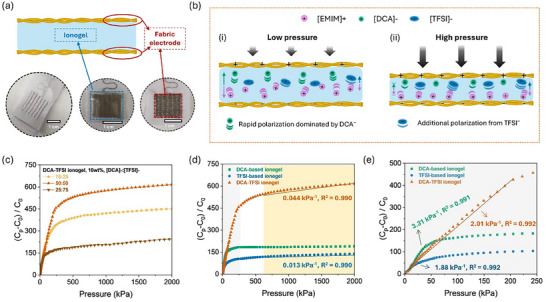
(a) Schematic illustration and optical images of the ionogel‐based capacitive pressure sensor showing the laminated structure of ionogel dielectric and fabric electrodes. (b) Schematic illustration of pressure‐dependent ionic polarization behavior in the DCA–TFSI ionogel. (c) Pressure‐dependent capacitive responses of DCA‐TFSI ionogels with different anion ratios (75:25, 50:50, 25:75). Comparison of the pressure‐dependent capacitive responses of DCA‐, TFSI‐, and DCA‐TFSI ionogels (d) up to 2000 kPa and (e) in the enlarged low‐pressure region (0–250 kPa), showing linear response fitting (thickness: 250 µm).

The capacitive response is evaluated using a sandwich‐structured sensor composed of a fabric electrode and an ionogel dielectric layer (Figure 2a). Frequency‐dependent capacitance measurements were first performed to examine the dielectric response of the ionic system (Figure S2). Based on these results, all capacitance–pressure measurements were conducted at 1 kHz, where stable dielectric response with minimal electrode polarization was observed. This configuration ensures intimate interfacial contact and uniform pressure transmission, providing a reliable platform for assessing dielectric behavior. The ionic‐liquid concentration and film thickness are optimized in single‐ion ionogels to establish a reference for comparison. Both DCA‐ and TFSI‐based ionogels exhibit clear concentration‐dependent behavior (Figures S3 and S4). The DCA‐based ionogel shows the highest sensitivity at 7.5 wt.%, attributed to the small and mobile DCA^−^ anions that promote rapid charge redistribution but also lead to early saturation under compression. In contrast, the TFSI‐based ionogel maintains gradual and stable polarization up to 10 wt.%, owing to the bulkier and more polarizable TFSI^−^ anions. Thinner films (≈250 µm) generate larger capacitance variations than thicker ones, consistent with shorter ionic migration paths and stronger effective electric fields. When the IL content exceeds the optimal concentration, both ionogels show reduced capacitance changes, indicating that excessive ionic loading weakens the effective dielectric field due to increased ion motion under pressure. Maintaining a balanced ionic fraction is therefore essential for consistent capacitive performance.

To examine the role of structural engineering in pressure sensing, the influence of surface morphology on capacitive performance was also examined. The DCA‐based ionogel was prepared using a sandpaper‐templating method to introduce random microscale roughness. The micro‐structured ionogel exhibited higher capacitive sensitivity in the low‐pressure region compared to the pristine flat sample, but the linear sensing range remained unchanged (Figure S5a). Linear fitting in the initial pressure regime yields a sensitivity of 2.85 kPa^−1^ (R^2^ = 0.99) for the pristine sample (0–20 kPa) and 5.00 kPa^−1^ (R^2^ = 0.99) for the sandpaper‐templated sample (0–15 kPa). These results confirm that structural modification substantially enhances low‐pressure responsiveness, but does not significantly alter the pressure range over which dielectric saturation occurs. Repeated loading–unloading cycles at low pressures (5, 10, and 30 kPa) showed stable and reproducible responses, confirming mechanical robustness (Figure S5b). Additionally, in practical demonstrations such as finger touch, palm pressing, finger bending, and arm bending, the sensor maintained consistent signal output under all conditions (Figure S5c–f). These results are consistent with previous reports indicating that surface roughness primarily enhances interfacial contact and charge responsiveness without expanding the linear operating range [55, 56]. In contrast, our findings indicate that the achievable linear pressure window is primarily governed by the dielectric composition rather than geometric modification. Therefore, to further extend the linear pressure range, we next explored an ion‐pair tuning approach by combining DCA^−^ and TFSI^−^ within a single polymer network.

To clarify the ion‐pair‐tuned dielectric behavior, Figure 2b illustrates the proposed polarization mechanism. At low pressure, the dielectric response is expected to be primarily influenced by the more mobile DCA^−^ anions, whereas at higher compression, contributions from the more strongly associated TFSI^−^ anions may become increasingly relevant. This proposed redistribution of polarization contributions is consistent with the observed delay in dielectric saturation and extension of the linear sensing regime.

To establish the appropriate IL concentration for the ion‐pair‐tuned system, we compared the 7.5 and 10 wt.% formulations of the 50:50 DCA‐TFSI ionogel. As shown in Figure S6, the 10 wt.% sample provides a larger and more stable capacitance change across the pressure range, whereas the 7.5 wt.% sample exhibits reduced responsiveness. Accordingly, 10 wt.% was selected as the representative ionic‐liquid content for all subsequent ion‐pair‐tuned ionogels.

For consistent comparison across compositions, capacitance–pressure measurements were carried out using ionogel films with a thickness of 250 µm and a 1.5 × 1.5 cm^2^ electrode area, unless otherwise noted. The capacitance–pressure response depends strongly on the [DCA]^−^:[TFSI]^−^ ratio (Figure 2c). Among the three tested compositions (75:25, 50:50, and 25:75), the 50:50 ionogel delivers the highest overall capacitance and the most uniform pressure response. This balanced combination of fast‐polarizing DCA^−^ and field‐stabilizing TFSI^−^ anions produces the most desirable dielectric behavior; hence, we refer to this formulation as the DCA–TFSI ionogel [57, 58]. The DCA–TFSI ionogel exhibits markedly improved sensing performance compared with its single‐ion counterparts (Figure 2d,e; detailed analyses in Figure S7). The DCA‐based ionogel shows a steep initial increase but saturates quickly, while the TFSI‐based ionogel responds more slowly yet remains stable under high pressure. In contrast, the ion‐pair‐tuned system maintains a continuous and nearly linear capacitance increase across the entire pressure window, achieving a high‐pressure sensitivity of 0.044 kPa^−1^ (R^2^ = 0.990) from 600 to 2000 kPa and excellent linearity (R^2^ = 0.992) up to 200 kPa. This broadened linear range demonstrates the effectiveness of ion‐pair tuning in mitigating dielectric saturation and stabilizing capacitive behavior.

### 2.3 Sensing Reliability and Environmental Stability

**FIGURE 3:**
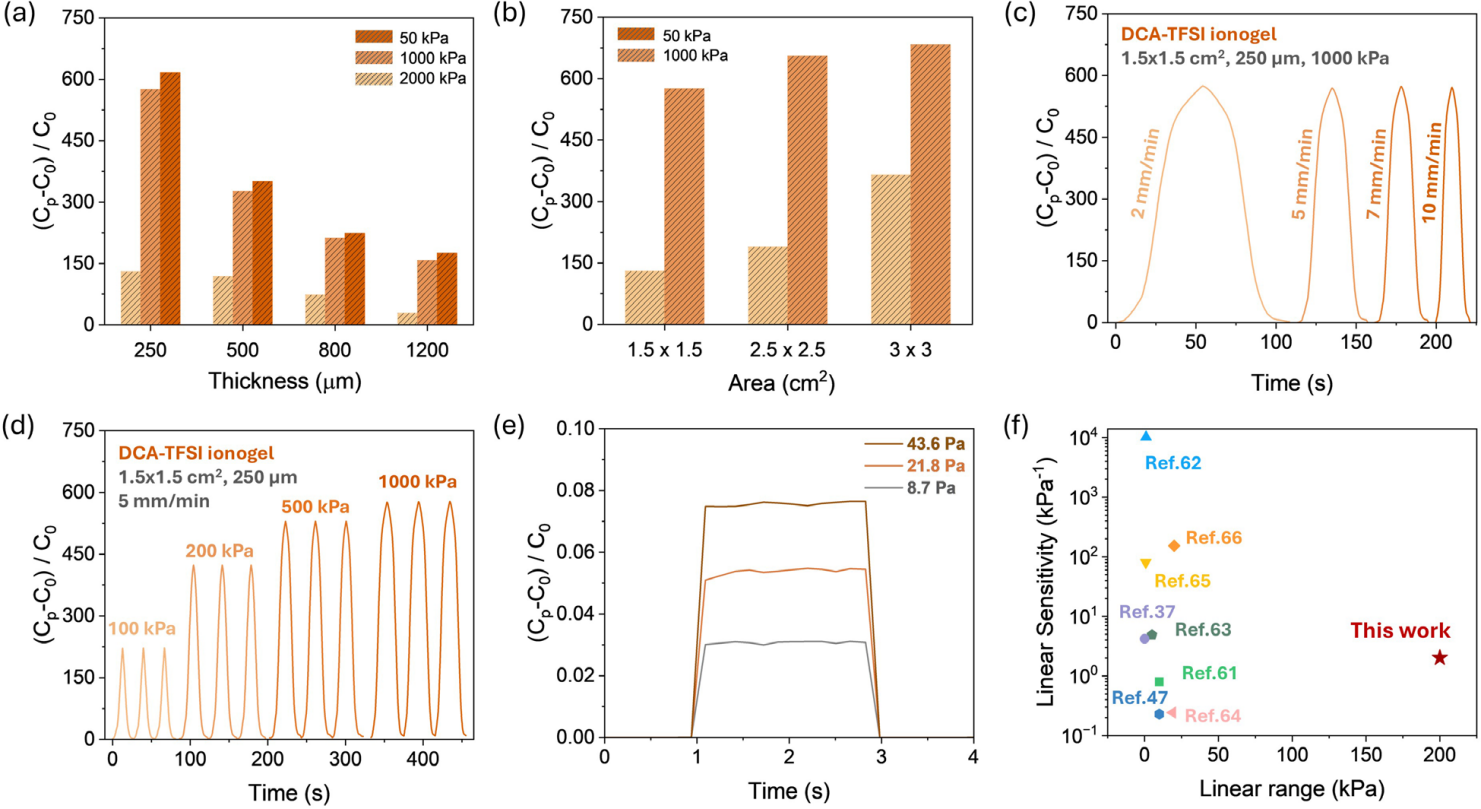
(a) Capacitance variations as a function of ionogel thickness. (area: 1.5 × 1.5 cm^2^) (b) Capacitance variations as a function of electrode area. (250 µm) (c) Response curves under different loading rates. (d) Cyclic stability test under repeated pressure loading at various magnitudes. (e) Detection of ultralow pressures down to 8.7, 21.8, and 43.6 Pa. (f) Comparison of linear sensitivity and sensing range with previously reported ionogel‐based capacitive sensors.

To further evaluate practical device performance beyond dielectric composition, we examined the influence of device geometry and dynamic loading conditions. Variations in device dimensions also affect the sensing characteristics of the DCA–TFSI ionogel (Figure 3a,b and Figure S8). Thinner films yield larger capacitance variations under the same pressure, likely due to shorter ionic migration distances and stronger electric‐field modulation during compression [59]. Larger electrode areas exhibit higher capacitance changes because pressure‐induced mechanical deformation and interfacial polarization accumulate over a wider active region—effects that are not fully canceled by the ΔC/C_0_ normalization [60].

The DCA–TFSI ionogel exhibits stable capacitive responses across different loading rates (Figure 3c). The nearly overlapping curves indicate that dielectric polarization occurs quickly enough to track externally applied pressure, confirming negligible rate dependence. The sensor also demonstrates strong repeatability during cyclic loading at various magnitudes (Figure 3d); the capacitance returns fully to baseline after each unloading cycle with minimal hysteresis. Long‐term cycling further highlights excellent durability, maintaining consistent performance after 10,000 loading cycles (Figure S9). The baseline capacitance shows only a small variation of approximately 7.63% over 10 000 cycles, indicating stable dielectric behavior under prolonged operation. Collectively, these results confirm that the ion‐pair‐tuned ionogel operates in a rate‐independent and fatigue‐resistant manner suitable for continuous, real‐world sensing applications.

The DCA–TFSI ionogel also maintains stable capacitive behavior under mechanical deformation (Figure S10a). The sensor operates reliably under tensile strains up to ∼30%, while larger strains cause thinning and eventual fracture (∼750 and 200 kPa for 30% and 50% strain, respectively). These results show that the ionogel preserves both mechanical integrity and dielectric stability under deformation levels relevant to wearable and prosthetic systems. Thermal stability is likewise robust: the capacitance–pressure response remains nearly unchanged between –20°C and 120°C (Figure S10b). Even after prolonged exposure at 80°C for 24–120 h (Figure S10c), the ionogel retains its original dielectric characteristics without noticeable degradation, reflecting the nonvolatile and thermally stable nature of the ionic‐liquid components.

The DCA–TFSI ionogel further demonstrates reliable operation in the ultralow‐pressure regime. Distinct and reproducible capacitance changes appear at 8.7, 21.8, and 43.6 Pa (Figure 3e), confirming high sensitivity to subtle physiological pressure variations. Compared with previously reported ionogel‐based capacitive sensors, this system provides a substantially broader linear range (≈200 kPa) while maintaining comparable sensitivity (Figure 3f) [37, 47, 61, 62, 63, 64, 65, 66]. A comprehensive comparison across representative dielectric material systems (Table S2) further indicates that many microstructured soft dielectrics achieve high sensitivity mainly within narrow pressure windows (typically <25–50 kPa), whereas the DCA–TFSI ionogel maintains stable linear capacitance–pressure behavior over an extended pressure range without requiring complex structural microengineering. Overall, these results demonstrate that ion‐pair tuning enables both low‐pressure detectability and high‐pressure durability, establishing the DCA–TFSI ionogel as a robust dielectric platform for integrated healthcare monitoring.

### 2.4 Demonstration of Prosthetic Adaptor Interface Pressure Monitoring

Maintaining appropriate pressure at the prosthetic adaptor interface is critical for prosthetic users, as localized overloading can cause discomfort and, over time, skin irritation [67, 68]. During walking, interface pressures typically range from several tens to a few hundred kilopascals, depending on body weight and socket fit [69, 70]. Sensors for this application must therefore provide stable and repeatable signals under moderate, cyclic mechanical loading rather than only under idealized static conditions. Conventional film‐ or board‐type sensors often fail to conform to the curved prosthetic adaptor and can exhibit signal drift under repeated loading and unloading [11, 71]. The ion‐pair‐tuned ionogel, which maintains stable capacitive responses across a broad pressure range up to 2000 kPa, offers a distinct advantage for these dynamic conditions. To evaluate its practical performance, ionogel‐based capacitive sensors are integrated onto a curved prosthetic adaptor to test their behavior under realistic, cyclic loading representative of daily use.

**FIGURE 4:**
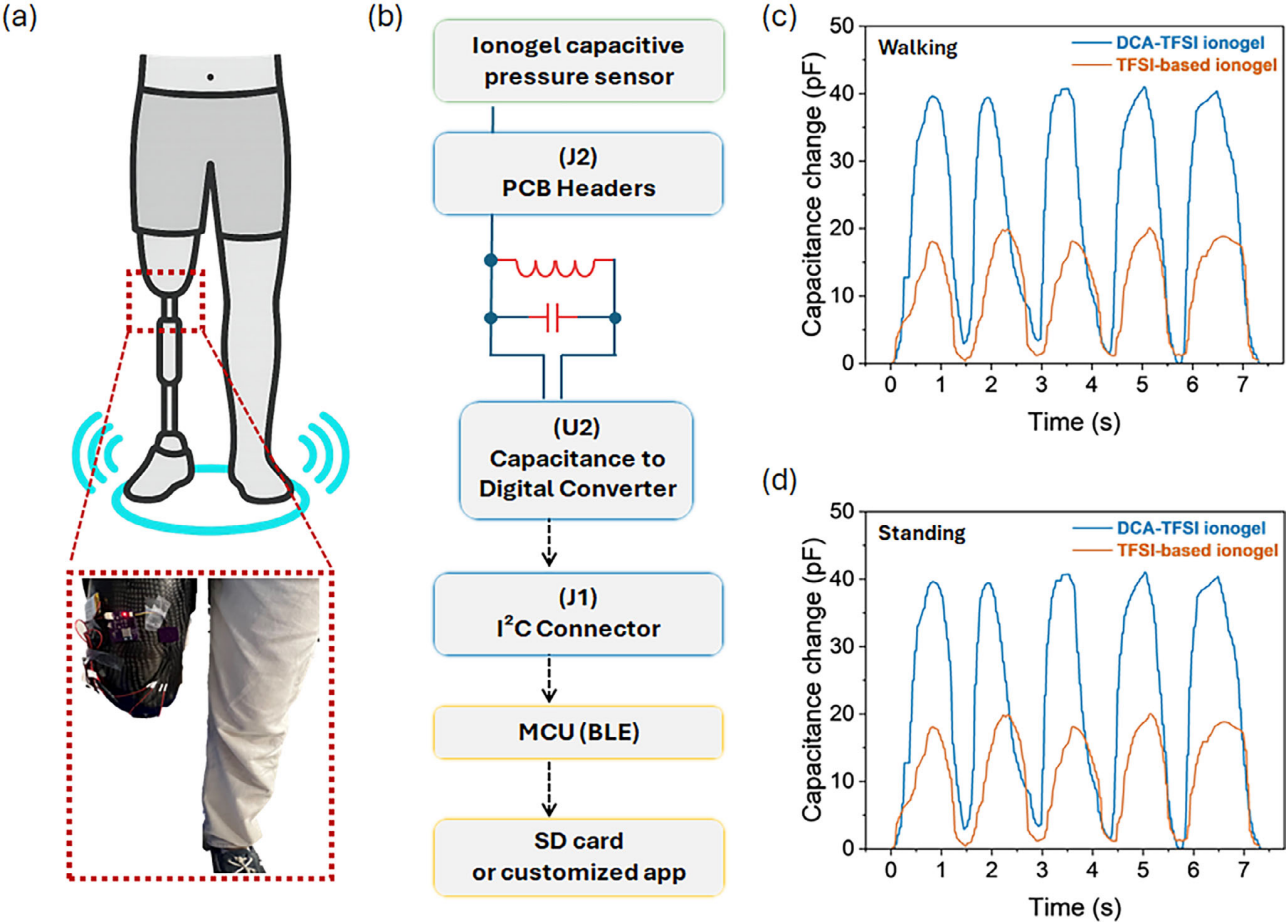
Demonstration of DCA‐TFSI ionogel‐based capacitive sensors for prosthetic adaptor interface pressure monitoring. (a) Schematic illustration and photograph of the sensor integrated on a carbon‐fiber prosthetic socket worn around the knee region. (b) Circuit diagram of the wireless data acquisition system consisting of a capacitance‐to‐digital converter, Bluetooth‐enabled microcontroller, and SD card module. (c) Dynamic pressure monitoring during walking and (d) static pressure monitoring during standing.

The sensors are mounted around the knee‐side region of the adaptor to emulate the mechanical stresses typically encountered during prosthetic motion (Figure 4a). This setup reproduces dynamic pressure variations while maintaining a safe, noninvasive testing configuration. The sensors are connected to a compact readout circuit consisting of a capacitance‐to‐digital converter, a Bluetooth‐enabled microcontroller, and an SD‐card module for real‐time data acquisition (Figure 4b). This portable system enables continuous monitoring of pressure changes during movement without altering the socket structure.

The DCA–TFSI ionogel exhibits distinct and periodic capacitance variations synchronized with gait‐like motion (Figure 4c). The response amplitude is higher than that of the TFSI‐based ionogel, indicating enhanced dielectric responsiveness under dynamic loading. Under static standing, the DCA–TFSI ionogel maintains a stable capacitance plateau with negligible drift or noise (Figure 4d), confirming reliable signal retention during prolonged loading. In addition, as shown in Figure S10a, the capacitance–pressure characteristics remain consistent under applied tensile strains up to 30%, indicating that moderate mechanical deformation during prosthetic motion does not compromise sensing performance. These device‐level results demonstrate that the ion‐pair‐tuned dielectric maintains its balanced sensitivity and linearity under realistic mechanical conditions. Collectively, the DCA–TFSI ionogel provides stable, repeatable, and high‐pressure‐tolerant capacitive sensing suitable for prosthetic adaptor interface evaluation and other wearable healthcare applications.

### 2.5 Demonstration of IOP Monitoring

Continuous monitoring of IOP is vital for glaucoma management, as the disease remains a leading cause of irreversible blindness [72]. Because IOP fluctuates with physiological and postural changes, single‐point clinical measurements often fail to capture transient variations essential for accurate diagnosis [73]. Conventional tonometers require direct corneal contact and trained operation, while most wearable ocular sensors rely on rigid components or embedded electronics that limit comfort and biocompatibility [45, 74]. These challenges underscore the need for soft, conformal sensors capable of stable and repeatable pressure detection within the physiological IOP range.

**FIGURE 5:**
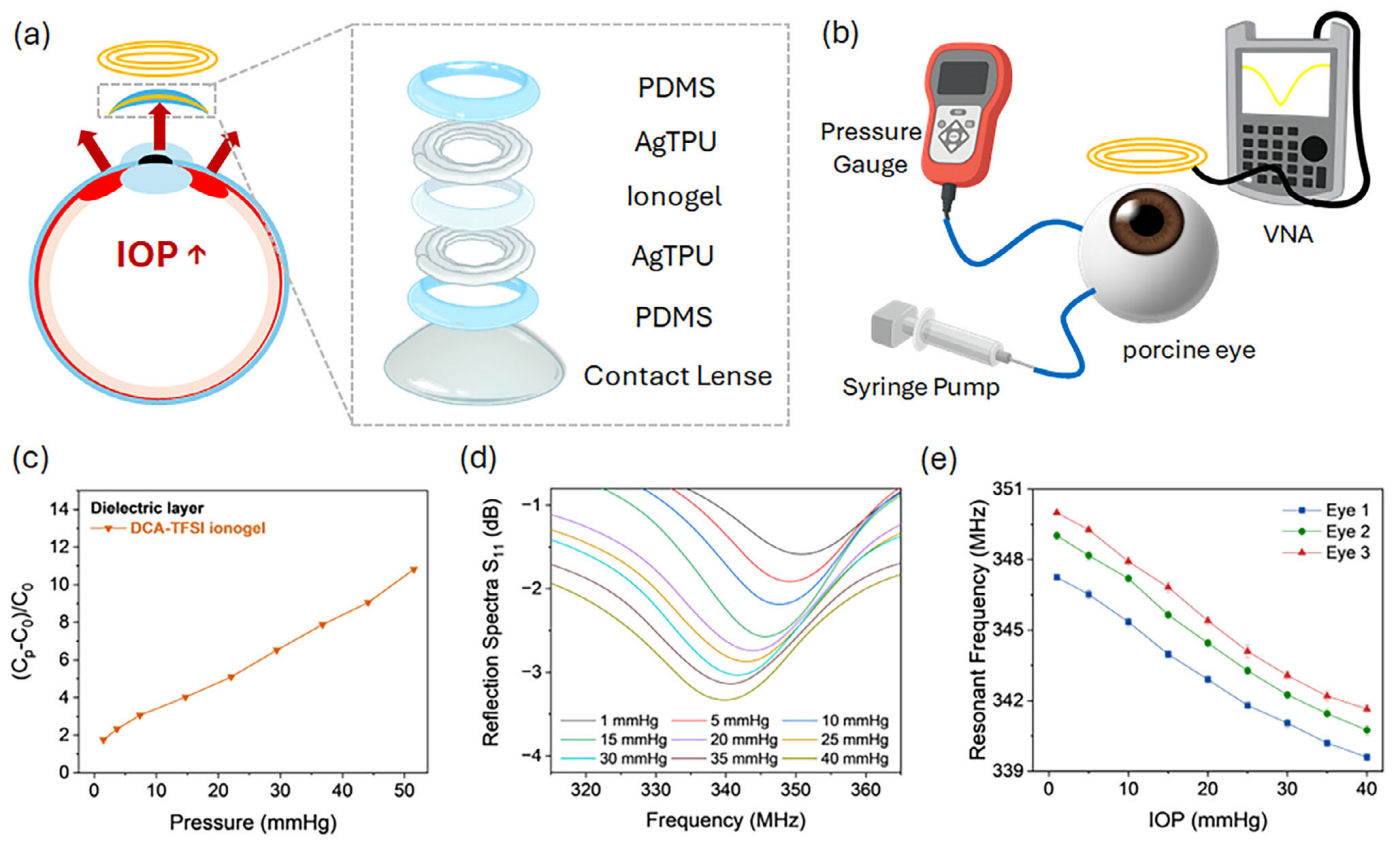
Demonstration of DCA‐TFSI ionogel‐based capacitive sensors for intraocular pressure (IOP) monitoring. (a) Schematic illustration of the multilayered lens‐integrated sensor composed of PDMS, AgTPU, and DCA‐TFSI ionogel dielectric layers. (b) Experimental setup for ex vivo porcine‐eye measurements using a syringe pump, pressure gauge, and vector network analyzer (VNA). (c) Reflection spectra (S_11_) of the wireless resonant circuit at different IOP levels from 1 to 40 mmHg. (d) Resonant frequency shifts as a function of applied pressure, showing consistent and repeatable responses across three different eyes. (e) Resonant frequency‐IOP curves measured from three porcine eyes.

To demonstrate the low‐pressure sensing capability of the DCA‐TFSI ionogel, we integrate it into a capacitive contact‐lens sensor designed for IOP monitoring (Figure 5a). The multilayer configuration—PDMS/AgTPU/ionogel/AgTPU/PDMS—is sequentially patterned onto a soft commercial contact lens via dispenser‐assisted printing, forming a flexible and conformal dielectric stack that maintains stable contact with the corneal surface. To ensure biocompatible interfacing with ocular tissue, the ionogel layer is fully encapsulated within PDMS outer layers, preventing direct contact between the ionic components and the corneal surface. Although imidazolium‐based ionic liquids have been reported to exhibit structure‐ and concentration‐dependent cytotoxicity [75, 76], the present device employs a moderate ionic‐liquid loading (10 wt.%) confined within a crosslinked polymer network and PDMS encapsulation to limit potential biological exposure. In addition, a skin irritation assessment based on hemoglobin mapping [77, 78] was conducted to evaluate material safety (Figure S11 and Note S1). No significant increase in hemoglobin content was observed for PDMS, ionogel, or PDMS‐encapsulated ionogel compared to untreated skin (*p* > 0.05), indicating negligible irritation under the tested conditions. Together, these results support the practical biocompatibility of the device architecture for wearable and ocular‐adjacent applications. The device is tested ex vivo using porcine eyes connected to a syringe pump and a pressure gauge to precisely control and monitor IOP levels (Figure 5b). The ionogel exhibits a linear increase in capacitance from 0 to ≈50 mmHg (Figure 5c), reflecting stable dielectric polarization across the physiological range. The corresponding wireless resonance‐based readout shows a continuous downshift in resonant frequency with increasing pressure (Figure 5d), resulting from geometric deformation of the LC circuit. These results confirm that the ionogel‐based contact lens transduces subtle IOP variations into measurable frequency shifts without signal distortion.

The sensor demonstrates consistent and reproducible performance across multiple biological samples (Figure 5e). The resonant frequency‐IOP curves from three porcine eyes follow similar linear trends, confirming reliable signal transduction with minimal inter‐sample variation. A comparative study between the DCA‐TFSI and DCA‐based ionogels (Figure S12) further reveals that both materials exhibit linear and reproducible capacitance changes within 0–50 mmHg, with the DCA‐based ionogel showing slightly higher capacitance variation. The ion‐pair‐tuned formulation, however, provides improved dielectric stability suited for continuous low‐pressure sensing. In addition, the device maintains stable wireless communication under realistic alignment conditions, showing minimal frequency drift (<1 MHz) when the coil angle (θ) is below 30° and the distance (d) is within 7 mm (Figure S13).

## 3 Conclusion

We develop an ion‐pair‐tuned ionogel that balances ionic polarization and suppresses dielectric saturation by integrating [EMIM][DCA] and [EMIM][TFSI] within a single polymer network. The combination of the highly mobile DCA^−^ and the strongly polarizable TFSI^−^ anions within a single polymer network enables synchronized charge redistribution and delays dielectric saturation, forming the physical basis for improved linearity. As a result, the DCA–TFSI ionogel exhibits enhanced dielectric linearity and stability across a broad pressure range, expanding the linear sensing window by nearly an order of magnitude—from ≈20 kPa in single‐ion systems to ≈200 kPa—while maintaining stable sensitivity of 2.01 kPa^−1^ (R^2^ = 0.992) at low pressures (≤200 kPa) and a consistent linear response of 0.044 kPa^−1^ (R^2^ = 0.990) between 600 and 2000 kPa. The material further demonstrates rate‐independent, fatigue‐resistant, and thermally stable capacitive responses under repeated deformation, underscoring its reliability for durable operation under repeated cycles.

The versatility of this ionogel is validated across distinct pressure regimes. In prosthetic adaptor interface sensing, the sensor produces reliable and repeatable signals under cyclic high‐pressure loading, confirming its suitability for wearable applications. In intraocular pressure monitoring, the same material detects subtle pressure variations within the physiological range, verifying its capability for continuous, low‐pressure healthcare monitoring. Together, these results establish ion‐pair‐tuned ionogels as an effective materials‐design strategy for achieving robust and linear dielectric behavior in soft electronic sensors.

The UV‐curable formulation supports conformal patterning through scalable printing or templating, facilitating integration with flexible and curved device architectures. The same ion‐pair tuning principle may extend to other soft‐electronic platforms that depend on controlled interfacial polarization, including piezocapacitive, electrophysiological, and multimodal self‐powered systems. Future studies will explore alternative ion‐pair combinations and polymer backbones to further tailor dielectric and mechanical properties, while assessing biocompatibility and durability over extended use for potential implantable or continuous biomedical applications.

## 4 Experimental Section

### 4.1 Materials

Benzyl acrylate (BA, stabilized with MEHQ, ≥97%) was obtained from TCI Chemicals (USA). Poly(ethylene glycol) methyl ether methacrylate (PEGMA, M_n_ ≈ 950), poly(ethylene glycol) diacrylate (PEGDA, M_n_ ≈ 700), and diphenyl(2,4,6‐trimethylbenzoyl)phosphine oxide (TPO) were purchased from Sigma‐Aldrich (USA). The ionic liquids 1‐ethyl‐3‐methylimidazolium bis(trifluoromethylsulfonyl)imide ([EMIM][TFSI], ≥98%) and 1‐ethyl‐3‐methylimidazolium dicyanamide ([EMIM][DCA], ≥98.0%, metals basis) were also obtained from Sigma‐Aldrich (USA). Thermoplastic polyurethane (TPU, Elastollan C60AW) was obtained from BASF, and silver flakes (2–5 µm) were purchased from Inframat Advanced Materials, Inc. All chemicals were used as received without further purification.

### 4.2 Synthesis of Ionogel and Sensor Fabrication

Ionogels were synthesized via UV‐initiated polymerization using benzyl acrylate (BA) and poly(ethylene glycol) methyl ether methacrylate (PEGMA) as the base monomers. Poly(ethylene glycol) diacrylate (PEGDA, 1 wt.%) and diphenyl(2,4,6‐trimethylbenzoyl)phosphine oxide (TPO, 1 wt.%) were added as the crosslinker and photoinitiator, respectively [47]. Two ionic liquids, [EMIM][DCA] and [EMIM][TFSI], were incorporated to prepare both single‐ion and ion‐pair‐tuned ionogels. For the single‐ion formulations, either [EMIM][DCA] or [EMIM][TFSI] was added individually at concentrations of 0, 2.5, 5, 7.5, 10, and 15 wt.% relative to the total monomer weight. For the ion‐pair‐tuned ionogels, the total ionic‐liquid content was fixed at 10 wt.%, and the molar ratio between [EMIM][DCA] and [EMIM][TFSI] was adjusted to 75:25, 50:50, and 25:75. All precursor solutions were magnetically stirred until clear and homogeneous, then poured into a mold and cured under 365 nm UV light for 5 min to form ionogel films. After curing, the resulting ionogel films were gently detached from the mold and directly integrated with textile‐based conductive electrodes to construct soft capacitive pressure sensors. The lamination process was performed under light mechanical pressure to ensure conformal contact between the ionogel and the fabric surface.

### 4.3 Characterization of Pressure Sensor

The electro‐mechanical performance of the ionogel‐based pressure sensors was evaluated using a precision LCR meter (Keysight E4980AL) operated under a two‐terminal configuration. Capacitance was measured at 1 kHz unless otherwise specified. Capacitance changes were recorded in real time through a customized LabVIEW‐based acquisition interface (National Instruments). Mechanical loading and unloading were applied using a universal testing platform (Mark‐10 ESM303, Willrich Precision Instruments) equipped with a 1 kN load cell.

### 4.4 Prosthetic Adaptor Interface Test Setup

To evaluate the performance of the ionogel‐based capacitive sensor under realistic loading conditions, the device was mounted on a curved prosthetic socket to simulate the limb–socket interface during use. The setup was designed to reproduce dynamic pressure variations that typically occur during sitting, standing, and walking‐like movements. The socket was worn to emulate mechanical loading representative of prosthetic use, without involving any human‐subject experiments or data collection. The sensor was connected to a portable data acquisition system consisting of a capacitance‐to‐digital converter (AD7746; Analog Devices), a Bluetooth‐enabled microcontroller (Adafruit nRF52840), and an SD‐card module for wireless recording.

### 4.5 Fabrication and Characterization of Ionogel‐Based Contact Lens Sensor

The ionogel‐based contact lens sensor was fabricated by sequentially stacking PDMS/AgTPU/ionogel/AgTPU/PDMS layers using a dispenser (Nordson EFD)‐assisted patterning process. The resulting multilayer structure formed a conformal and flexible dielectric stack compatible with ocular curvature. The electrical performance of the ionogel sensor was characterized using a precision LCR meter (Keysight E4980AL) under controlled compression to verify its capacitive response and baseline stability before ex vivo testing.

### 4.6 Ex Vivo Evaluation in Porcine Eyes

The ex vivo IOP response of the ionogel‐based contact lens was evaluated using freshly enucleated porcine eyes to replicate physiological IOP variations. Each eye was cannulated with two 30‐gauge needles: one connected to a microinjection pump (UltraMicroPump3 with Micro4 controller; World Precision Instruments) for controlled infusion and withdrawal of saline solution into the anterior chamber, and the other connected to a digital pressure manometer (HHP452; OMEGA Engineering) for real‐time pressure calibration. The ionogel‐based contact lens was gently placed on the corneal surface to ensure conformal contact, and an external reader coil was positioned within approximately 10 mm for optimal wireless coupling during measurement. The IOP was varied from 1 to 40 mmHg in 5 mmHg increments, and the resonant reflection spectra (S_11_) were continuously monitored using a handheld RF analyzer (N9913A FieldFox; Keysight Technologies). The resonant frequency shift was extracted at each step to evaluate the pressure‐dependent response. Measurements were repeated on three separate eyes to confirm reproducibility.

## Funding

This research was financially supported by the Ministry of Trade, Industry, and Energy (MOTIE) of Korea under the Global Industrial Technology Cooperation Center (GITCC) Program supervised by the Korea Institute for Advancement of Technology (KIAT) (Task No. P0028319) for the development of the ionogel. This research was also supported by Korean Institute for Advancement ofTechnology (KIAT) grant funded by the Korea Government (MOTIE) (RS‐2024‐00435157, Human Resource Development Program for IndustrialInnovation (Global)). Additional support was provided in part by the National Eye Institute of the National Institutes of Health under Award Number R01EY034901‐01A1 for the intraocular pressure sensing study and by the National Institute of Biomedical Imaging and Bioengineering (NIBIB) of the National Institutes of Health under Award Number R21EB034879 for the prosthetic sensing study.

## Conflicts of Interest

The authors declare no conflicts of interest.

## Supporting information




**Supporting file**: advs74758‐sup‐0001‐SuppMat.docx

## Data Availability

The data that support the findings of this study are available in the supplementary material of this article.
