# Supplementary material for: Ion‐Pair‐Tuned Ionogels for Broad‐Range Linear Pressure Sensing
Source: Adv Sci (Weinh). 2026 Mar 12;13(30):e24195. doi: 10.1002/advs.202524195 (PMC13248827; doi:10.1002/advs.202524195)
Supplement: Supplementary file 1 — Supporting file: advs74758‐sup‐0001‐SuppMat.docx [file ADVS-13-e24195-s001.docx]

Supporting Information

Ion-Pair-Tuned Ionogels for Broad-Range Linear Pressure Sensing

Hyeonseo Joo, Tianhao Yu, Yumin Dai, Seokkyoon Hong, Axel González Cornejo, Tristan Michael Long, Sang Mok Park, Pete S. Kollbaum, Edgar Bolívar-Nieto, Young L. Kim, Dong Rip Kim* and Chi Hwan Lee*

**Table S1.** Comparative summary of dielectric materials used in capacitive pressure sensors.

| Material Type | Sensitivity | Linear Pressure Range | Mechanical Stability | Environmental Stability | Remarks |
| --- | --- | --- | --- | --- | --- |
| Inorganic | ◎ | × | × | ○ | High permittivity but mechanically brittle and unsuitable for flexible systems |
| Elastomer (PDMS, TPU, etc..) | × | △ | ○ | ○ | Highly flexible but low permittivity and geometry-dependent deformation |
| Hydrogel | ◎ | △ | ○ | × | High ionic mobility but prone to dehydration, limiting long-term stability |
| Ionogel | ◎ | △ | ○ | ◎ | Stable but nonlinear under high pressure due to ion imbalance |
| This work | ◎ | ◎ | ○ | ◎ | Balanced ionic mobility and delayed dielectric saturation, enabling broad-range linear response |

^◎ Excellent ○ Good △ Moderate × Poor^

**Table S2.** Performance comparison of the DCA–TFSI ionogel with previously reported ionogel-based capacitive sensors.

|  | Ref. | Dielectric Material | Sensitivity (kPa^-1^) | Linear Range | Max Pressure (kPa) |
| --- | --- | --- | --- | --- | --- |
| Elastomer | ^[1]^ | PDMS (microdome) | 0.44% kPa^-1^ 0.0084% kPa^-1^ | 0-11 100-500 | 500 |
| Inorganic/Elastomer | ^[2]^ | Graphene/PS microspheres/PDMS | 0.209 0.004 | 0-3 25-150 | 150 |
|  | ^[3]^ | MWCNTs/PDMS (gradient porous composite) | 0.134 0.0098 | 0-200 200-400 | 400 |
|  | ^[4]^ | TPU-AgNPs/ PDMS (microstructured) | 0.091 0.001 | 0-5 300-2000 | 2000 |
| Hydrogel | ^[5]^ | porous PDMS skeleton and a SA Hydrogel | 14.25 0.31 | 20-50 200-500 | 500 |
|  | ^[6]^ | Ionic Hydrogels (microstructured) | 37.94 1.27 | 0-25 120-290 | 290 |
| Aerogel | ^[7]^ | Polyimide-based Aerogel | 1.41 0.70 | 0-40 400-200 | 200 |
|  | ^[8]^ | Cellulose/PVA/CNT Aerogel | 66.4 14.8 | 0-15 15-50 | 50 |
| Ionogel | This work | DCA-TFSI ionogel | 2.01 0.044 | 0-200 600-2000 | 2000 |

[1] E. Thouti, K. Chauhan, R. Prajesh, M. Farman, R. K. Maurya, P. Sharma, A. Nagaraju, *Sensors and Actuators A: Physical* **2022**, 335, 113393.

[2] X. Wu, W. Zhao, J. Duan, Z. Qu, J. Wang, B. Zhang, *Materials Letters* **2022**, 326, 132952.

[3] L. Chen, B. Zheng, S. Li, H. Wu, *Measurement* **2025**, 118359.

[4] X. Li, Y. Liu, Y. Ding, M. Zhang, Z. Lin, Y. Hao, Y. Li, J. Chang, *ACS Applied Materials & Interfaces* **2024**, 16, 12974.

[5] H. Huang, X. Ran, S. Wan, Y. Wang, H. Bi, *Nanoscale* **2024**, 16, 17926.

[6] C. Liu, F. Ma, Q. Sun, Q. Hu, W. Tong, X. Guo, R. Hu, P. Liu, Y. Huang, X. Hao, *ACS Applied Materials & Interfaces* **2024**, 16, 34042.

[7] M. Cheng, Y. Yuan, Q. Li, C. Chen, J. Chen, K. Tian, M. Zhang, Q. Fu, H. Deng, *Journal of Materials Science & Technology* **2025**, 217, 60.

[8] J. Cao, G. Sun, P. Wang, C. Meng, *ACS Applied Materials & Interfaces* **2024**, 16, 54652.





**Figure S1.** Chemical structures of monomers (BA, PEGMA, PEGDA) and ionic liquids ([EMIM][DCA], [EMIM][TFSI]), and schematic illustration of ionogel fabrication via UV-curing of the precursor solution.


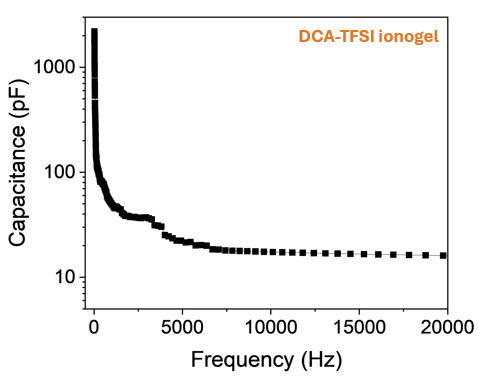


**Figure S2.** Frequency-dependent capacitance of the ionogel dielectric.





**Figure S3.** Pressure-dependent capacitive responses of DCA-based ionogels with varying ionic liquid concentrations (0-15 wt%) and film thicknesses of (a) 250 µm, (b) 500 µm, (c) 800 µm, and (d) 1200 µm.





**Figure S4.** Pressure-dependent capacitive responses of TFSI-based ionogels with varying ionic liquid concentrations (0-15 wt%) and film thicknesses of (a) 250 µm, (b) 500 µm, (c) 800 µm, and (d) 1200 µm.


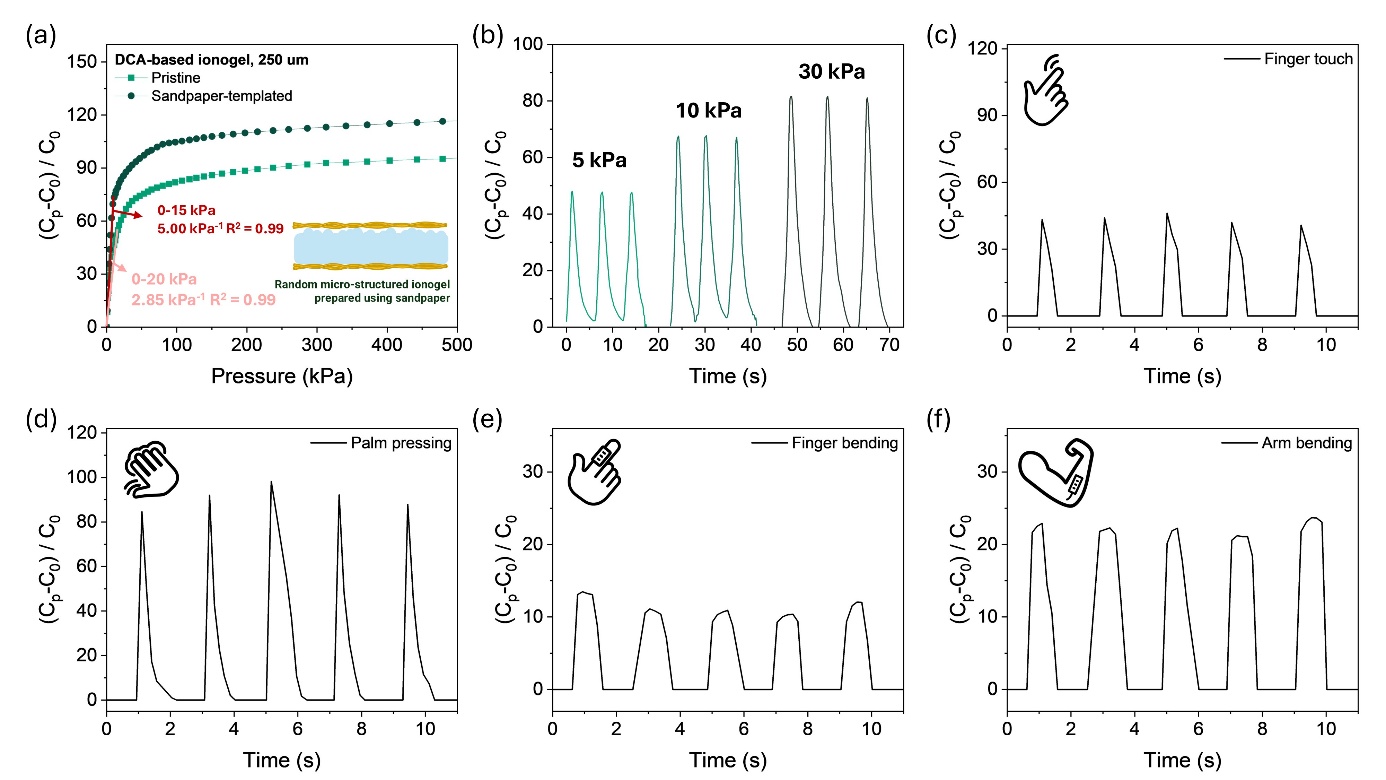


**Figure S5.** (a) Pressure-dependent capacitive responses of pristine and sandpaper-templated DCA-based ionogels (thickness: 250 µm; electrode area: 0.5 × 2 cm^2^). (b) Cyclic loading-unloading responses under low pressures (5, 10, and 30 kPa). Real-time capacitive responses of the sandpaper-templated ionogel under various mechanical stimuli: (c) finger touch, (d) palm pressing, (e) finger bending and (f) arm bending.





**Figure S6.** Comparison of capacitance–pressure behavior for 7.5 wt% and 10 wt% DCA-TFSI ionogels (50:50).





**Figure S7.** Linear fitting of capacitive response-pressure curves for (a) DCA-, (b) TFSI-, and (c) DCA-TFSI ionogels in the high-pressure range (150 - 2000 kPa), (d) DCA-, (e) TFSI-, and (f) DCA-TFSI ionogels in the high-pressure range (up to 200 kPa), analyzed for each pressure segment.

**

**

**Figure S8.** Full-range capacitive response–pressure curves of DCA-TFSI ionogels with (a) different film thicknesses and (b) different electrode areas corresponding to Figures 2d and 2e, respectively.


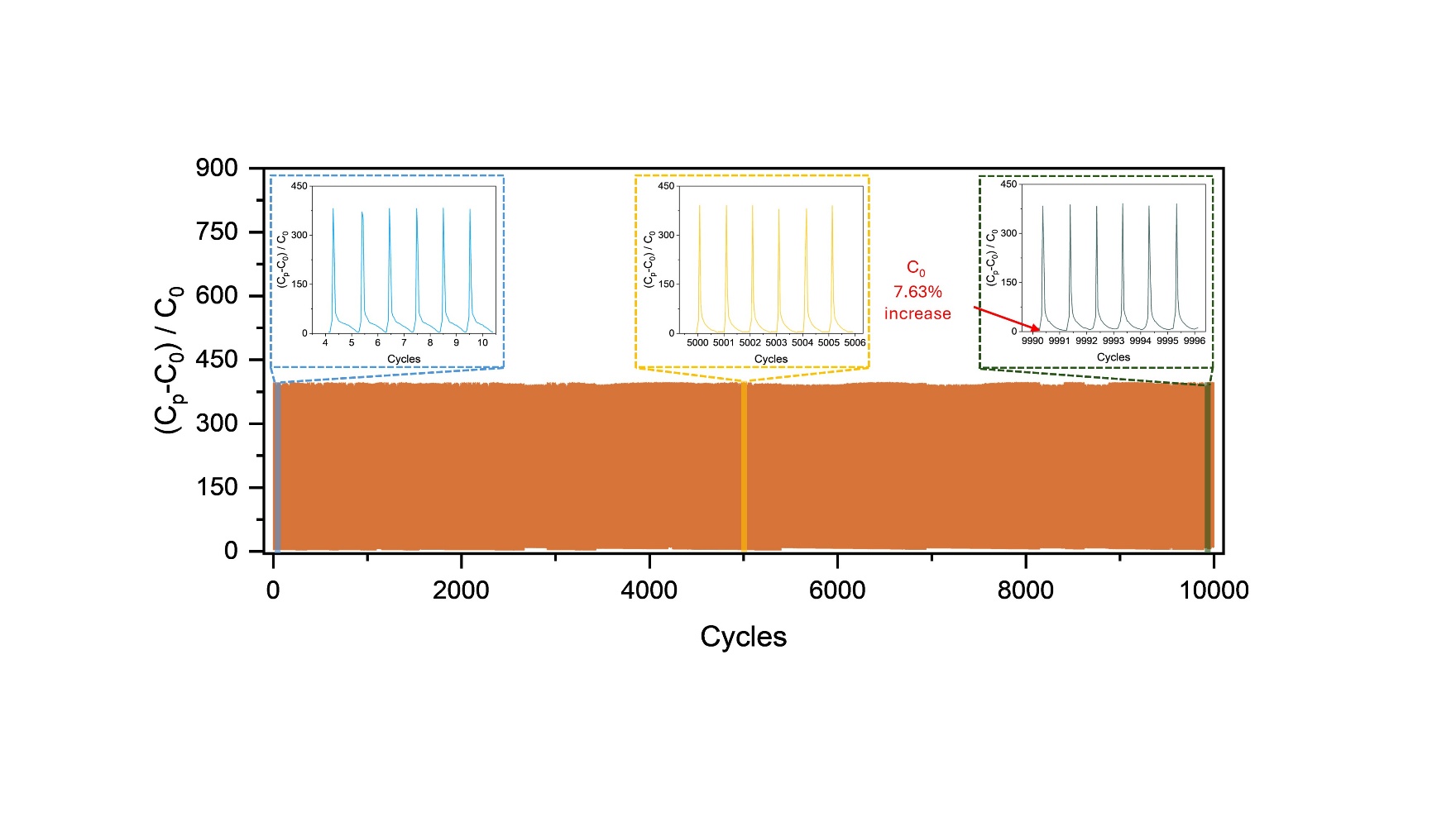


**Figure S9.** Long-term cyclic stability of the DCA–TFSI ionogel under repeated loading. The device maintains stable capacitive response over 10,000 cycles with a baseline capacitance variation of approximately 7.63%.


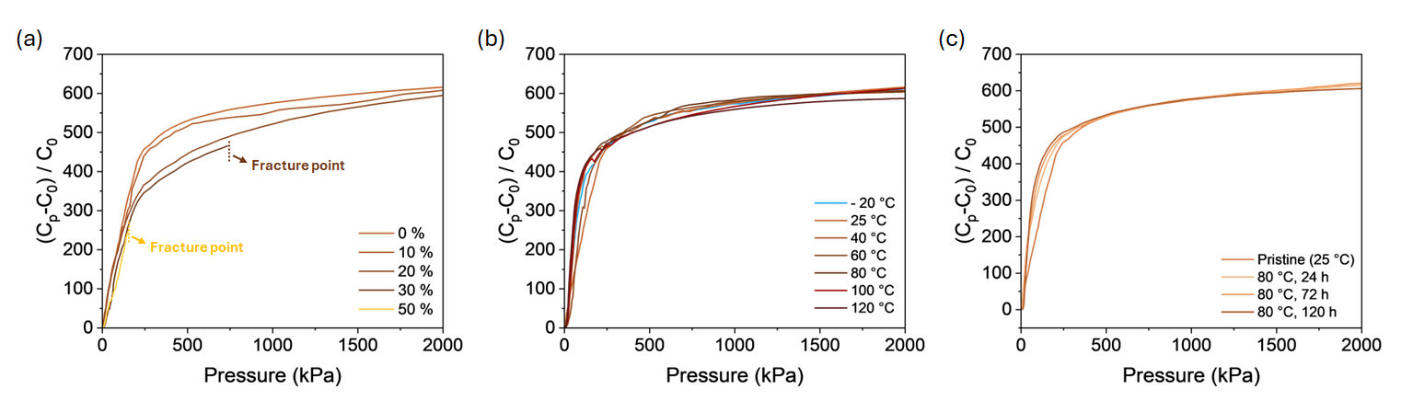


**Figure S10.** (a) Capacitive responses of DCA-TFSI ionogel under different tensile strains (0 - 50%). Capacitive responses of the DCA-TFSI ionogel under thermal stress conditions: (b) temperature-dependent responses from -20 °C to 120 °C and (c) stability after prolonged exposure at 80 °C for 24, 72, and 120 h.





**Figure S11.** Skin irritation assessment of PDMS, ionogel, and PDMS-encapsulated ionogel (PDMS+ionogel) (a) Representative RGB images and corresponding 2D hemoglobin (Hgb) maps after 10 min exposure (scale bar: 5 mm). (b) Quantified hemoglobin content (mean ± SD); no significant difference compared to control (p > 0.05).





**Figure S12.** Comparative evaluation of low-pressure IOP sensing performance between DCA-TFSI and DCA-based ionogels. (a) Capacitance variation with increasing pressure (0–50 mmHg). (b) Reflection spectra (S_11_) of the resonant circuit at different IOP levels. (c) Corresponding resonant frequency shifts as a function of IOP, showing comparable linearity and sensitivity for both ionogels.





**Figure S13.** Evaluation of wireless coupling stability between the reader coil and the lens-integrated DCA-TFSI ionogel sensor. (a) Frequency shift as a function of angular misalignment (θ) between the coils. (b) Frequency shift as a function of vertical distance (d) between the coils.

**Note S1. Skin irritation test**

To evaluate the biocompatibility of the materials, a skin irritation test was conducted by quantifying hemoglobin (Hgb) content using optical hemoglobin mapping. PDMS, ionogel, and PDMS-encapsulated ionogel samples were placed on human skin for approximately 10 min and then removed. Untreated bare skin served as a negative control.

The measured hemoglobin values were: control (3.18 ± 0.03 mg/mL), PDMS (3.18 ± 0.02 mg/mL), ionogel (3.12 ± 0.02 mg/mL), and PDMS-encapsulated ionogel (3.18 ± 0.02 mg/mL) (mean ± SD). A one-tailed Bonferroni-corrected post-hoc test confirmed no significant increase in hemoglobin content compared to the control (p > 0.05).

These results indicate that neither the ionogel nor the encapsulated device induces detectable skin irritation under the tested conditions.
